# Biomechanical effects of Evans versus Hintermann osteotomy for treating adult acquired flatfoot deformity: a patient-specific finite element investigation

**DOI:** 10.1186/s13018-024-04584-4

**Published:** 2024-02-01

**Authors:** Can Xu, Hua Liu, Mingqing Li, Hui Li, Chun’ang Pan

**Affiliations:** 1 Department of Orthopedics, National Clinical Research Center for Geriatric Disorders, Xiangya Hospital, Central South University, Changsha, 410008 Hunan People’s Republic of China; 2 Beijing Engineering and Technology Research Center for Medical Endoplants, Beijing, People’s Republic of China

**Keywords:** Flatfoot, Adult acquired flatfoot deformity, Finite element, Lateral column lengthening, Evans osteotomy, Hintermann osteotomy

## Abstract

**Background:**

Evans and Hintermann lateral column lengthening (LCL) procedures are both widely used to correct adult acquired flatfoot deformity (AAFD), and have both shown good clinical results. The aim of this study was to compare these two procedures in terms of corrective ability and biomechanics influence on the Chopart and subtalar joints through finite element (FE) analysis.

**Methods:**

Twelve patient-specific FE models were established and validated. The Hintermann osteotomy was performed between the medial and posterior facets of the subtalar joint; while, the Evans osteotomy was performed on the anterior neck of the calcaneus around 10 mm from the calcaneocuboid joint surface. In each procedure, a triangular wedge of varying size was inserted at the lateral edge. The two procedures were then compared based on the measured strains of superomedial calcaneonavicular ligaments and planter facia, the talus-first metatarsal angle, and the contact characteristics of talonavicular, calcaneocuboid and subtalar joints.

**Results:**

The Hintermann procedure achieved a greater correction of the talus-first metatarsal angle than Evans when using grafts of the same size, indicating that Hintermann had stronger corrective ability. However, its distributions of von-Mises stress in the subtalar, talonavicular and calcaneocuboid joints were less homogeneous than those of Evans. In addition, the strains of superomedial calcaneonavicular ligaments and planter facia of Hintermann were also greater than those of Evans, but both generally within the safe range (less than 6%).

**Conclusion:**

This FE analysis study indicates that both Evans and Hintermann procedures have good corrective ability for AAFD. Compared to Evans, Hintermann procedure can provide a stronger corrective effect while causing greater disturbance to the biomechanics of Chopart joints, which may be an important mechanism of arthritis. Nevertheless, it yields a better protection to the subtalar joint than Evans osteotomy.

**Clinical relevance:**

Both Evans and Hintermann LCL surgeries have a considerable impact on adjacent joints and ligament tissues. Such effects alongside the overcorrection problem should be cautiously considered when choosing the specific surgical method.

**Level of evidence:**

Level III, case–control study.

**Supplementary Information:**

The online version contains supplementary material available at 10.1186/s13018-024-04584-4.

## Introduction

Adult acquired flatfoot deformity (AAFD) is a common deformity with manifestations including hindfoot valgus, forefoot abduction, and midfoot varus, while its historical nomenclature is still confusing. As a widely used technique to correct AAFD, lateral column lengthening (LCL) surgeries are mainly recommended when the amount of talonavicular (TN) joint non-coverage area is more than 40% [1]. There are multiple specific procedures for LCL, of which Evans and Hintermann osteotomies are most commonly adopted in clinical practice [2, 3], but neither has yet been proven more successful than the other [4, 5]. The Evans osteotomy is usually performed at a position slightly more than 10 mm proximal to the calcaneocuboid (CC) joint [5]. Due to a close distance to the CC joint, some researchers argued that the Evans procedure would destabilize the anterior calcaneus, provoking the incongruency and increase in pressure in the CC joint [6]. In contrast, the Hintermann osteotomy is performed close to the anterior border of the posterior subtalar facet, therefore concentrating on the TN joint axis, which is considered to be the main center of rotation of the subtalar joint complex [7]. Theoretically, Hintermann should exert a less impact on the CC joint. Nonetheless, there is still no comparative study focusing on the orthotic ability and biomechanics influence of LCL procedures on the Chopart and subtalar joints when using grafts of the same size, so further biomechanical research is warranted to confirm aforementioned assumptions.

Traditional biomechanical studies are mostly carried out using cadaver models, making it difficult to acquire the strain data of soft tissues and contact characteristics [8]. In this regard, computer simulation based on finite element (FE) analysis is able to obtain internal biomechanical information such as soft tissue strain and joint contact characteristics under predetermined conditions and controlled environment. Therefore, it can effectively and efficiently evaluate a series of variables (such as surgical techniques and various pathologies) to optimize the protocol design, screening, prediction, and treatment in orthopedics [9]. The aim of this study was to compare the corrective ability and biomechanics influence between Evans and Hintermann osteotomy procedures using a previously validated FE model [10].

## Materials and methods

**Fig. 1:**
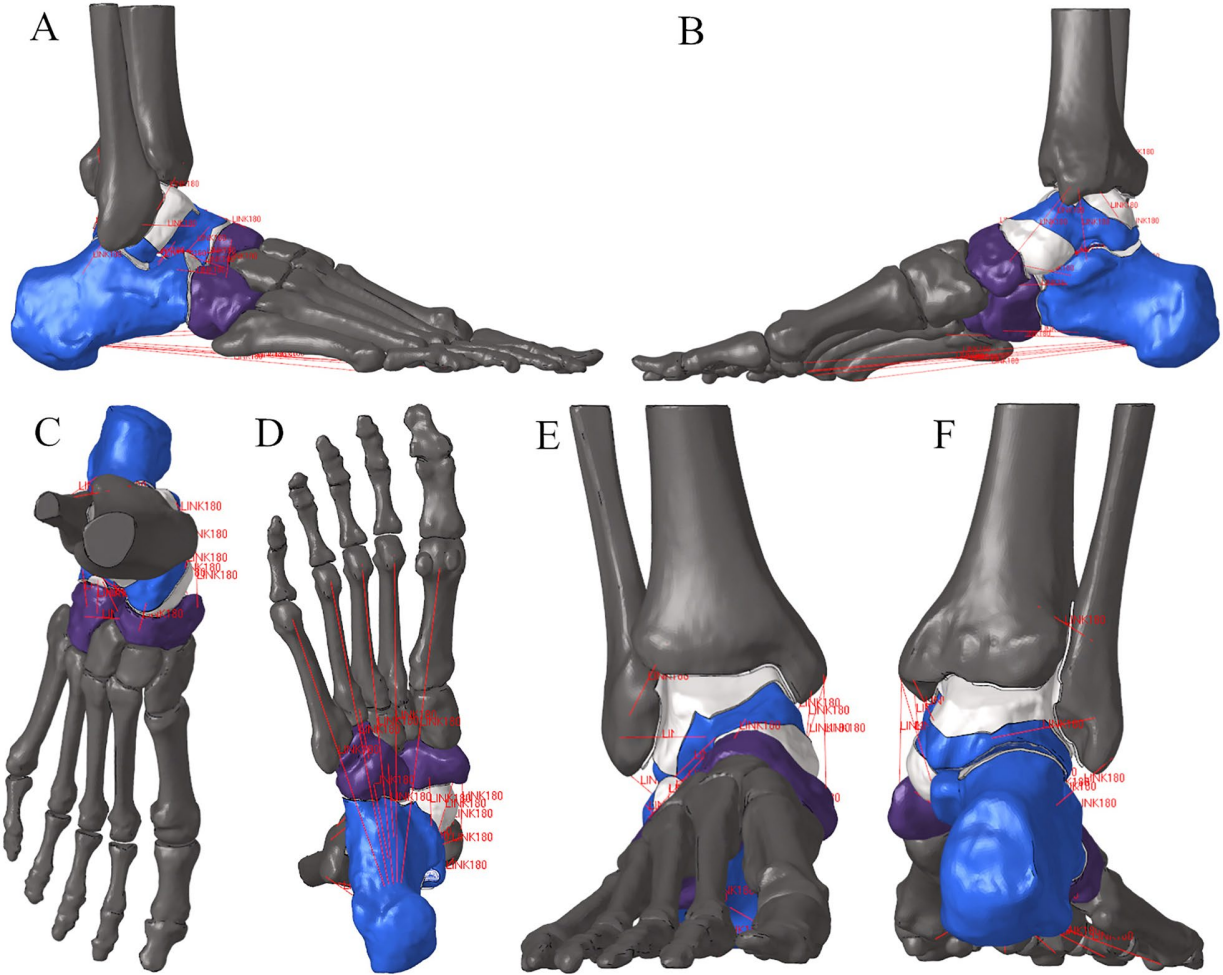
FE model: **A**, **B** lateral and medial view of the foot, **C**, **D** top and plantar view of the foot, **E**, **F** anterior and posterior view of the ankle. The red lines represent different ligaments

The present study had been approved by the Ethic Committee of the authors’ institutions. Data were derived from 12 volunteers with flexible flatfoot deformity (stage IIb) recruited in our previous research [10]. Patient-specific three-dimensional FE models of the foot and ankle joints (Fig. 1) were created in Abaqus/Explicit (Abaqus, Pawtucket, RI). The contours of the entire bone structure of the foot and ankle (including the distal tibia and fibula) and the articular cartilage were derived from MRI images (Fig. 1), and the geometry of these structures was reconstructed using Mimics 17.0 (Materialize Software, Belgium). The extracted surface was then smoothed and refined using Geomagic Studio 2013 (Geomagic, USA). Since the smoothing operation tends to substantially reduce the FE-predicted contact stress by 20–30% in peak contact stress [11], for successful contact analysis, the smoothing of the cartilage was kept as minimal as possible. The average 3D deviation between the original cartilage surface and the smooth surface was 0.27 mm.

FE meshes were obtained using commercial preprocessing software (Hypermesh, Altair Corporation, Troy, MI), and the number of elements was determined by a mesh convergence test. Mesh convergence was considered if the average variation in ligament stress between subsequent meshes was less than 5%. The relevant material parameters in the model are detailed in Additional file 1: Supplementary tables 1–3. Specifically, the bony structure was meshed by two-dimensional rigid triangular shell element, while the joint cartilage was meshed by three-dimensional tetrahedral deformable element and fixed to the shell element of the bony structure. The ligaments were simulated with one or multiple tension only link elements. The spring ligament (SL) and planter fascia (PF) were captured through arrays of multiple elements to depict the major bands of these structures. The insertion and original positions of the ligaments were determined based on both MRI images and the reported anatomical positions. The cartilage was modeled as an isotropic, homogeneous, linear elastic structure with the Young's modulus of 12 MPa and Poisson's ratio of 0.42 [10].

To create accurate AAFD models for each individual case, MRI images were analyzed by an experienced radiologist to determine the degree of soft tissue degeneration. The soft tissues of the medial column, such as SL and PF, were graded according to their signal attenuation and were assigned with a reduced stiffness value [12]. The cartilage–bone and cartilage–cartilage interfaces were defined by the tied and sliding contact algorithms. The contact behavior between joint surfaces was treated as frictionless.

Numerical simulation was performed using ABAQUS/Explicit (ABAQUS, Pawtucket, RI) software. The initial position of the model was determined by the relaxed foot ankle position of the patient during MRI examination. In this study, simulation of the neutral position of the foot ankle was achieved in Hypermesh by specifying the translation and rotation of the foot. Therefore, prior to the simulation, the neutral weight-bearing position was prescribed by an experienced foot and ankle expert first, and each model was contrasted with the foot standing anteroposterior (AP) and lateral radiographs to obtain accurate positioning. During this process, no stress would be formed on the ankle ligaments, so the stress of ankle ligaments was set to zero. Subsequently, a physiological load was applied to the FE model to simulate the midstance phase during gait. The weight of the patient was applied vertically to the tibial axis, and at the same time, linear elements were added to the model to provide further support for bony constraints. The muscle tendon activation values were treated as a tension only structure. Five muscles, namely Achilles tendon, flexor hallucis longus, peroneus longus, peroneus brevis, and flexor digitorum longus tendons, were selected for simulation and were assigned with a static load of 50%, 10.5%, 10%, 8.8%, and 6% of the body weight, respectively [10]. The posterior tibial tendon was excluded from the loading protocol as its dysfunction is the hallmark of stage IIb AAFD [13]. And the anterior tibialis was also excluded for its relatively less important in midstance phase. Ultimately, each model was created based on the specific anatomy and body weight of the patient. After applying the body weight and activating muscle forces, all the models were equilibrated to their final state.

**Fig. 2:**
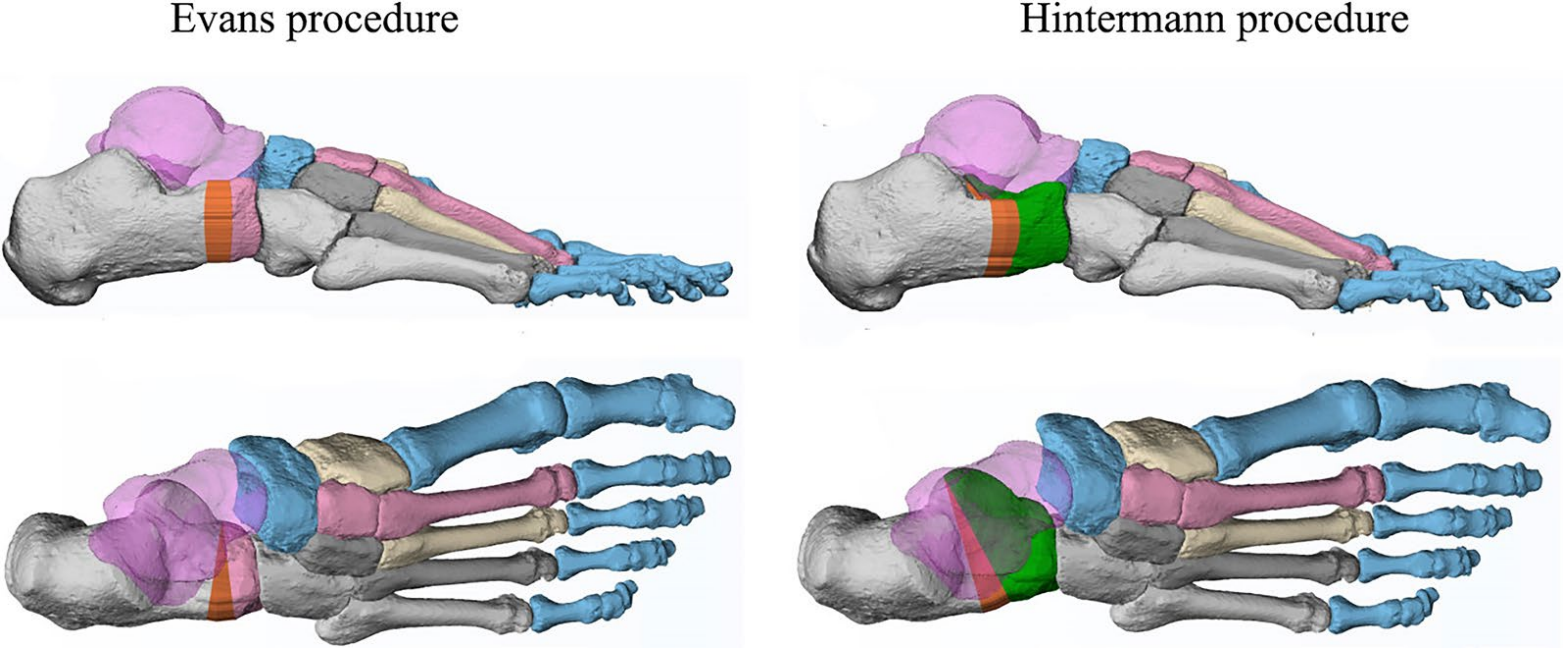
Simulation of the Evans and Hintermann osteotomy with triangular grafts

In the simulation process, the Hintermann osteotomy was performed between the medial and posterior facets of the subtalar joint; while, the Evans osteotomy was created on the anterior neck of the calcaneus around 10 mm from the CC joint surface (Fig. 2). Then, a triangular wedge of a varying size was inserted at the lateral edge in each surgery. The specific size (lateral length) of the wedge ranged from 2 to 14 mm, with an increment of 2 mm (i.e., 2, 4, 6, 8, 10, 12, and 14 mm).

The insertion of the wedge would produce disturbance between the hindfoot and midfoot. To eliminate such gaps, adduction was performed on the midfoot and forefoot (around the axis formed at the medial edge of the surgery) to create a new TN joint coverage, while maintaining the relative relationship of the bones. Then, the bony structures distal to the Chopart joint were rotated in dorsiflexion (around the axis on the dorsal side of the navicular) to close the plantar gap at the Chopart joint, and the distal bones were moved proximally to further close the residual gap. Eventually, the FE model was equilibrated to its final state. Throughout this simulation process, the tension generated by the ligaments would ultimately determine the physiological bone position. To comparatively analyze the biomechanical effects exerted by wedges of different sizes, the angular measurements, contact stresses of joints, and ligament strains were quantified in all molds.

**Fig. 3:**
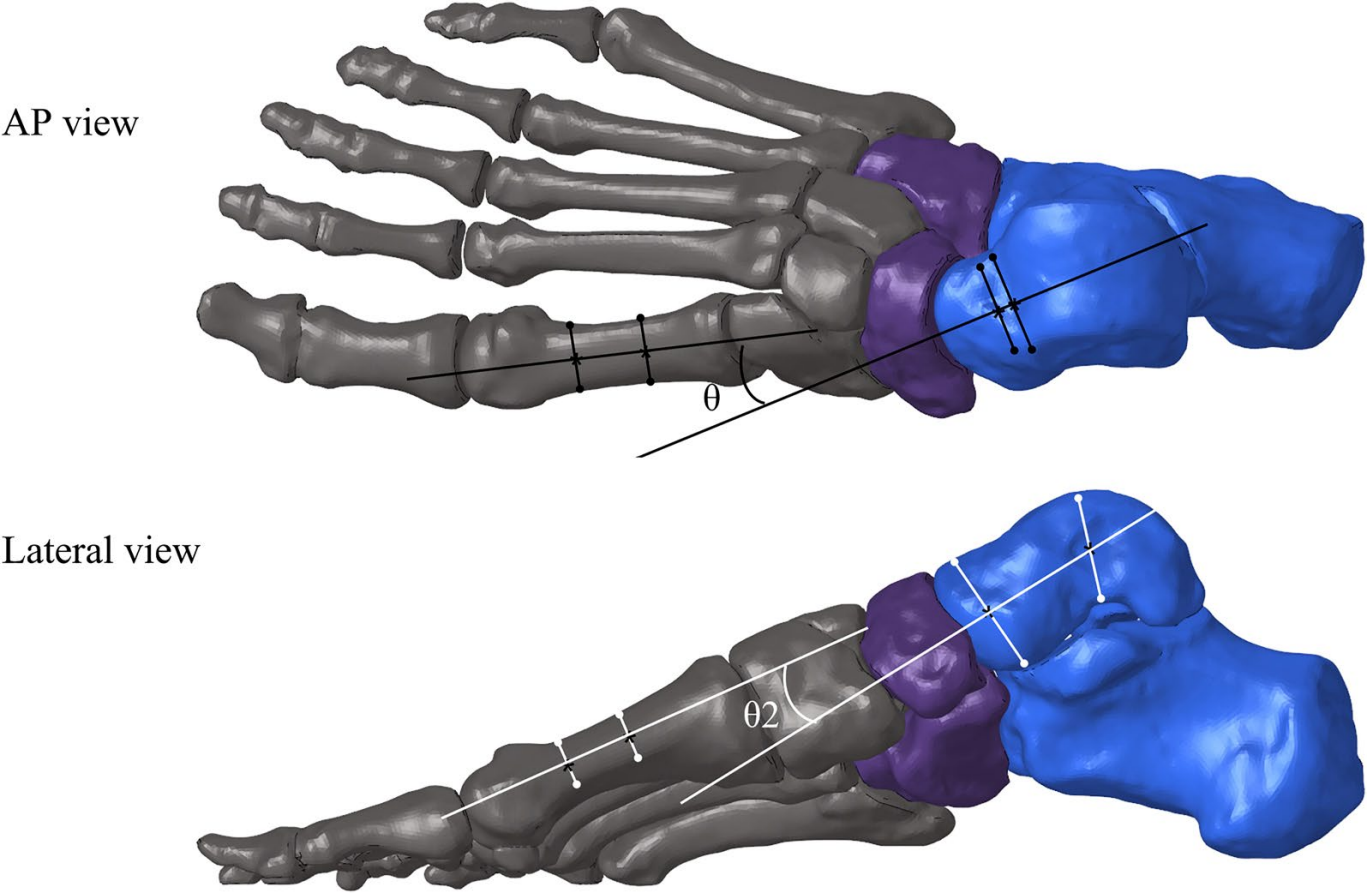
Talus-first metatarsal angle in AP and lateral view

A previous study found that PF and SL were the main tissues supporting the arch; while, plantar ligaments played a secondary role [14]. The SL was constituted by the superomedial calcaneonavicular (SMCN) and inferior calcaneonavicular ligaments. The strains of SMCN ligaments, and the medial (calcaneus to the first metatarsal) and lateral parts (calcaneus to the fifth metatarsal) of PF were determined respectively when the equilibrium state was achieved. The contact characteristics of CC, TN, and subtalar joints were then calculated accordingly. In addition, the talus-first metatarsal angle (Tal-1MA) in the AP and lateral views was also measured (Fig. 3), in order to assess the angle of internal rotation and arch correction [15].

To validate the FE model, distance and angular measurements were conducted to replicate the clinical radiographic measurements when the equilibration was acquired under the applied loading condition. The predicted values were compared between the model results and the results derived from radiographs, and the agreement between the two was quantified by linear regression. It was found that the R^2^ between the model and radiograph for the angel and distance measurements ranged from 0.276 to 0.884 [10]. Furthermore, when comparing with normal controls and the AAFD population, the means and standard deviations of the 12 subjects were markedly different from those of normal feet but similar to those of AAFD feet. Then, the average and peak pressures of TN and CC joints were compared with the published data, and the difference between the simulation and the published experimental results was found to be less than 10% [16–18]. Detailed results of verification test can be found in Additional file 1: Supplementary tables 4, 5. Lastly, a sensitivity study was carried out on the mechanical properties of the ligaments and cartilage. The parameter A and B of the curve fit data, as well as the Young’s modulus of the cartilage, were observed to vary within a range of ± 10%. The maximum difference in radiograph angle and distance measures was compared [10].

## Results

**Fig. 4:**
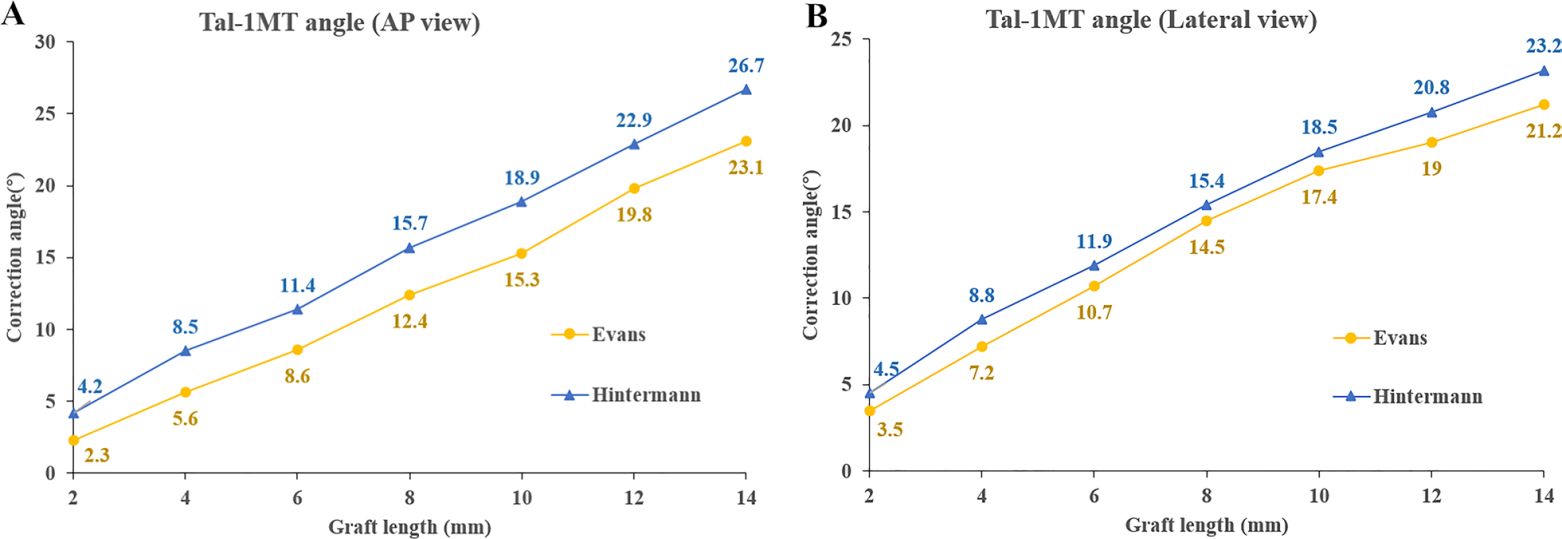
Talus-first metatarsal correction angle in AP and lateral views in two groups

In general, the angle of correction showed a growing trend with the increase in graft size in both Evans and Hintermann osteotomy procedures. The Tal-1MA in the AP and lateral views measured preoperatively was 25.3° ± 4.4° and 11.2° ± 2.7°, respectively. With the increase in wedge length, the correction angle gradually increased based on the data measured postoperatively (Fig. 4A, B), and that of the Hintermann procedure was found to be greater at the same graft size.

**Fig. 5:**
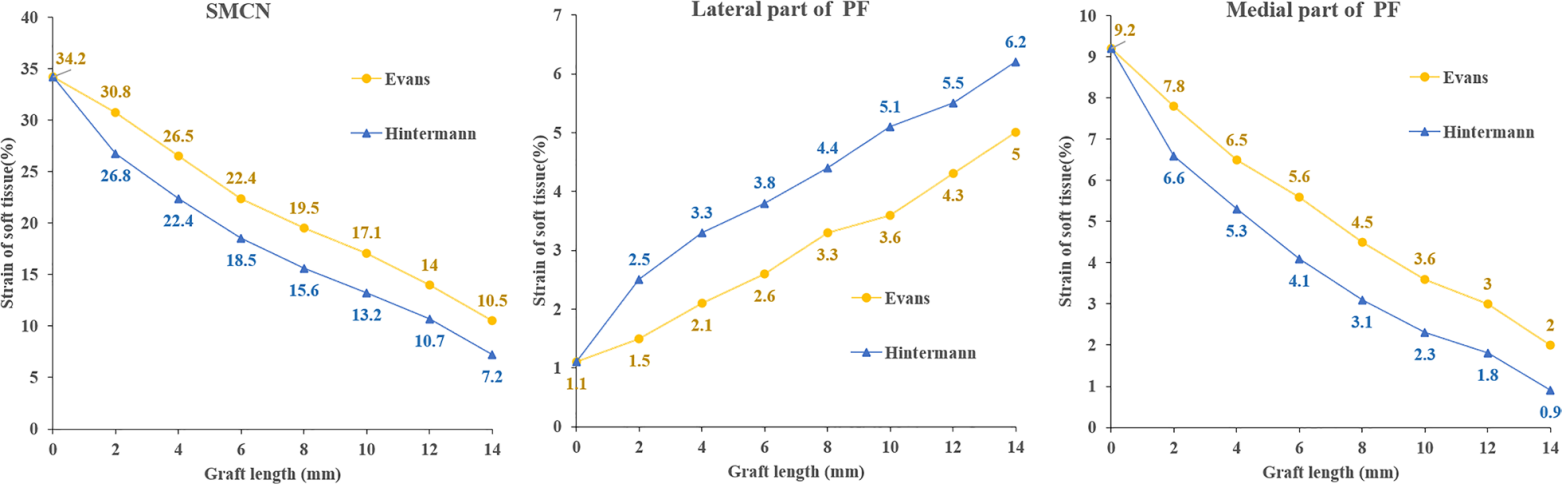
Strain of SMCN and PF in two groups

As shown in Fig. 5, the strain of the SMCN ligament exhibited a declining trend with the increase in graft length (Fig. 5a), and that of the Hintermann group was lower. Both surgical methods had an impact on the strain of the medial and lateral parts of the PF. Specifically, the strain at the medial part of the PF became smaller with the increase in graft length (Fig. 5c); whereas, the strain at the lateral part gradually increased (Fig. 5b). For the same graft size, the Hintermann procedure had a greater impact on the strain of the medial and lateral parts of the PF.

**Fig. 6:**
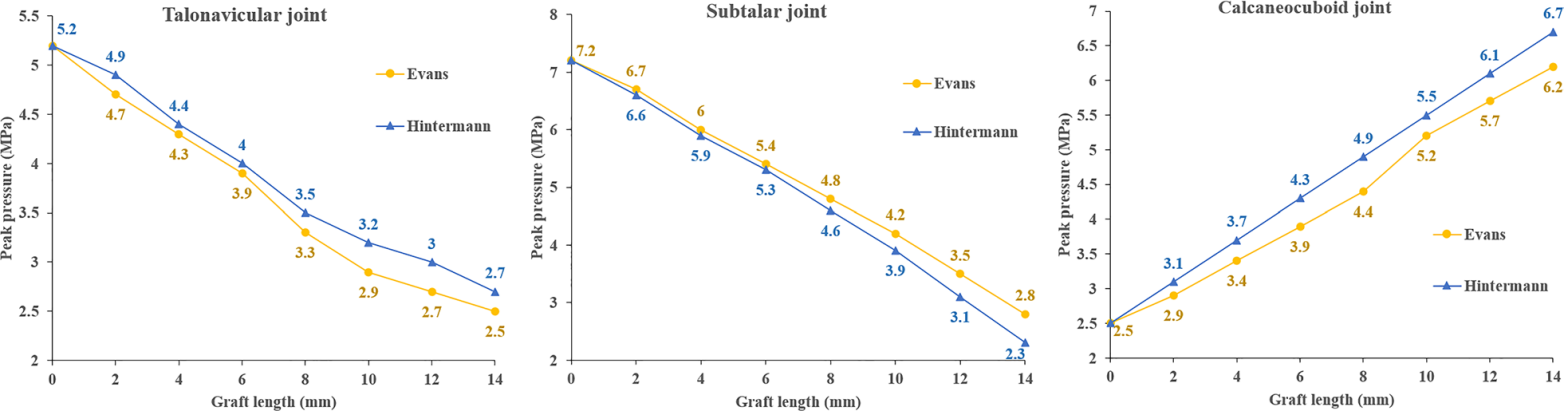
Mean peak pressure of TN, CC, and subtalar joints in two groups

As shown in Fig. 6, in both procedures, the average peak contact pressure of the TN and subtalar joints exhibited a declining trend with the increase in graft length (Fig. 6a, c), whereas that of the CC joint exhibited a growing trend (Fig. 6b). For the same graft length, the Hintermann procedure produced a greater peak pressure in the CC and TN joints.

**Fig. 7:**
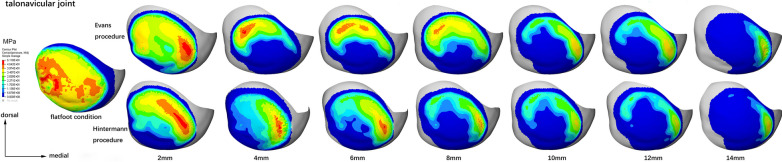
Contact stress distribution of TN joint in a typical model

**Fig. 8:**
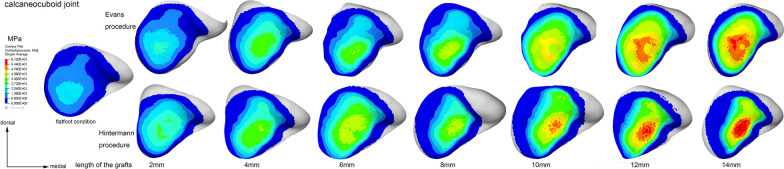
Contact stress distribution of CC joint in a typical model

**Fig. 9:**
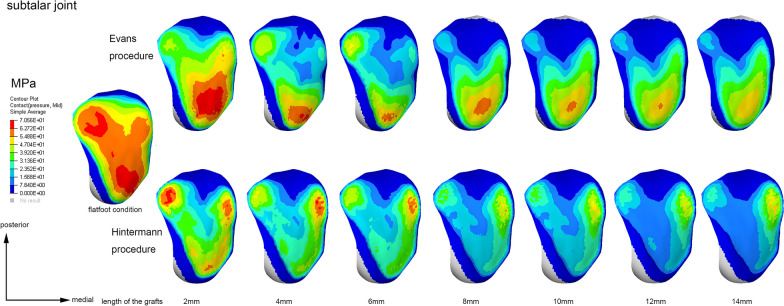
Contact stress distribution of subtalar joint in a typical model

The contact stress distribution characteristics of the TN, CC, and subtalar joints of a typical model are shown in Figs. 7, 8 and 9, respectively. As it can be seen, with the increase in graft size, the two centers of the TN joint pressure gradually shifted toward the medial region of the joint (Fig. 7). The peak pressure was higher and the contact area was smaller in the Hintermann group (Fig. 7). The stress center of the CC joint gradually shifted toward the dorsomedial region with the increase in graft size (Fig. 8). Similarly, the peak pressure was higher and the contact area was smaller in the Hintermann group (Fig. 8). For the subtalar joint, there were 3 peak pressure centers that were located in the lateral, medial and anterior regions of the posterior facet, respectively. With the increase in graft size, the pressure centers of the posterior subtalar joint gradually shifted toward the lateral region and eventually only 2 centers were left (Fig. 9). The peak pressure was lower and the contact area was smaller in the Hintermann group (Fig. 9).

## Discussion

In this FE study, we compared two different LCL procedures (i.e., Evans and Hintermann) in terms of their corrective ability and biomechanics influence on the Chopart and subtalar joints. The results suggested that the corrective ability of Hintermann was stronger than that of Evans when using grafts of the same length. However, the contact stress characteristics of the surrounding joints after Hintermann osteotomy appeared to be more abnormal compared to the Evans procedure.

The corrective ability of Hintermann and Evans procedures have been analyzed in previous studies. Chan JY et al. found that when the mean amount of Evans osteotomy performed was 7.4 ± 1.4 mm, the Tal-1MA on the AP X-ray films decreased from 19.3° (range − 4.9° ~ 38.8°) preoperatively to 8.5° (range − 15.6° ~ 24.4°) postoperatively, with a mean reduction of about 10.8° [2]. Similar results were obtained in our study. In the Evans group, the Tal-1MA (AP view) showed a correction angle of 8.6° ± 2.8° and 12.4° ± 2.3° corresponding to the graft length of 6 mm and 8 mm, respectively (Fig. 4a). Ettinger et al. compared the postoperative radiological outcomes of both osteotomies and found no significant differences between the two procedures [3]. However, the mean length of grafts used in the Evans procedure was bigger than that of the Hintermann procedure in their study (11.0 mm vs. 9.4 mm). Such results, in another way, suggest that the amount of correction of Tal-1MA after the Hintermann procedure is greater than that after Evans when using grafts of the same length.

Overcorrection is a severe problem in clinical practice when LCL surgery is performed, so the typical range of LCL recommended is 5–10 mm [19]. In our study, simulations were performed on triangular grafts ranging from 2 to 14 mm, with an increment of 2 mm, and the Tal-1MA measured postoperatively was compared with that in the normal population (7.1° ± 6.6° and 3.3° ± 4.7° for the AP and lateral view [20], respectively). If the corrected joint angle fell within 1 standard deviation of the normal value, the correction would be considered appropriate [20]. Our results reveal that adequate Tal-1MA correction (15.7° ± 1.7° and 15.4° ± 3.7° for the AP and lateral view, respectively) can be achieved by the Hintermann procedure once the graft length has reached 8 mm (Fig. 4). This finding is consistent with a previous cadaveric study, which reported that 6 mm Hintermann LCL restored the alignment closest to the intact foot; while, 10 mm lengthening tended to lead to overcorrection [21].

Malakoutikhah et al. investigated the effect of individual ligament tears on the changes in joint contact mechanics based on the peak pressure of the TN, CC and subtalar joints [22]. In our study, the mean peak pressure of aforementioned joints was 5.2 ± 0.4 MPa, 2.5 ± 0.5 MPa and 7.2 ± 1.1 MPa, respectively, which was similar to Malakoutikhah’s results [22]. The potential impact of LCL surgery on the surrounding joints is an issue deserving careful consideration. Kimberly et al. compared the pressure of the CC joint between Hintermann and Evans procedures in a cadaveric study [23], and found that the normalized mean and normalized peak CC pressures were significantly higher in the Evans group than in the Hintermann group at every lengthening level. However, their flatfoot model was created from normal cadaveric specimens, and the graft shape and the detailed technique of graft placement were unclear. In a clinical study by Ettinger et al. [3], the postoperative degenerative changes in the CC joint and subtalar joint were 41% and 18% in the Evans group, versus 25% and 14% in the Hintermann group, suggesting a higher rate of protection achieved by Hintermann for the CC and subtalar joints. However, the mean graft size for the Evans procedure (11.0 mm) was bigger than that of Hintermann (9.4 mm) in their study. Previous studies have found that the pressure of CC joint would increase significantly once the graft size exceeded 10 mm, thus tending to cause arthritis [24–26]. Thus, if the graft size remains the same (less than 10 mm), the degenerative change after the Evans procedure may be equivalent to or even less than that of Hintermann. Besides, the fixation method is another key factor to be considered [3]. In Ettinger’s study, headed screws were used in 88.2% of the Hintermann cases and 55.6% of the Evans cases. When using this type of implant, the screws are inserted at a position very close to the CC joint, which will elevate the possible risk of joint damage and subsequent irritation. After taking these known risk factors into account, the Hintermann procedure does not necessarily outperform Evans in terms of CC joint protection.

**Fig. 10:**
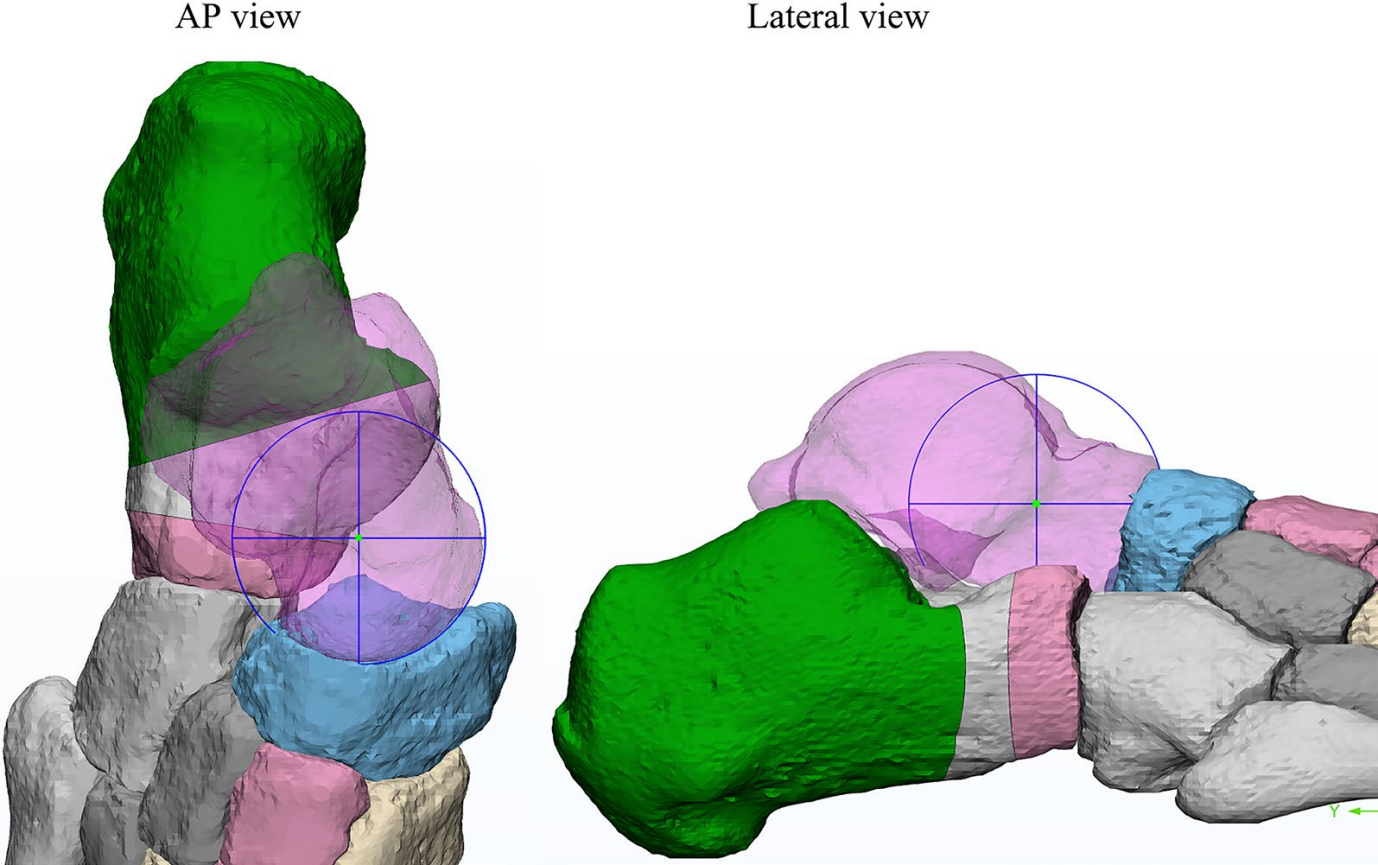
Rotation center of TN joint and both procedures (AP and lateral view)

**Fig. 11:**
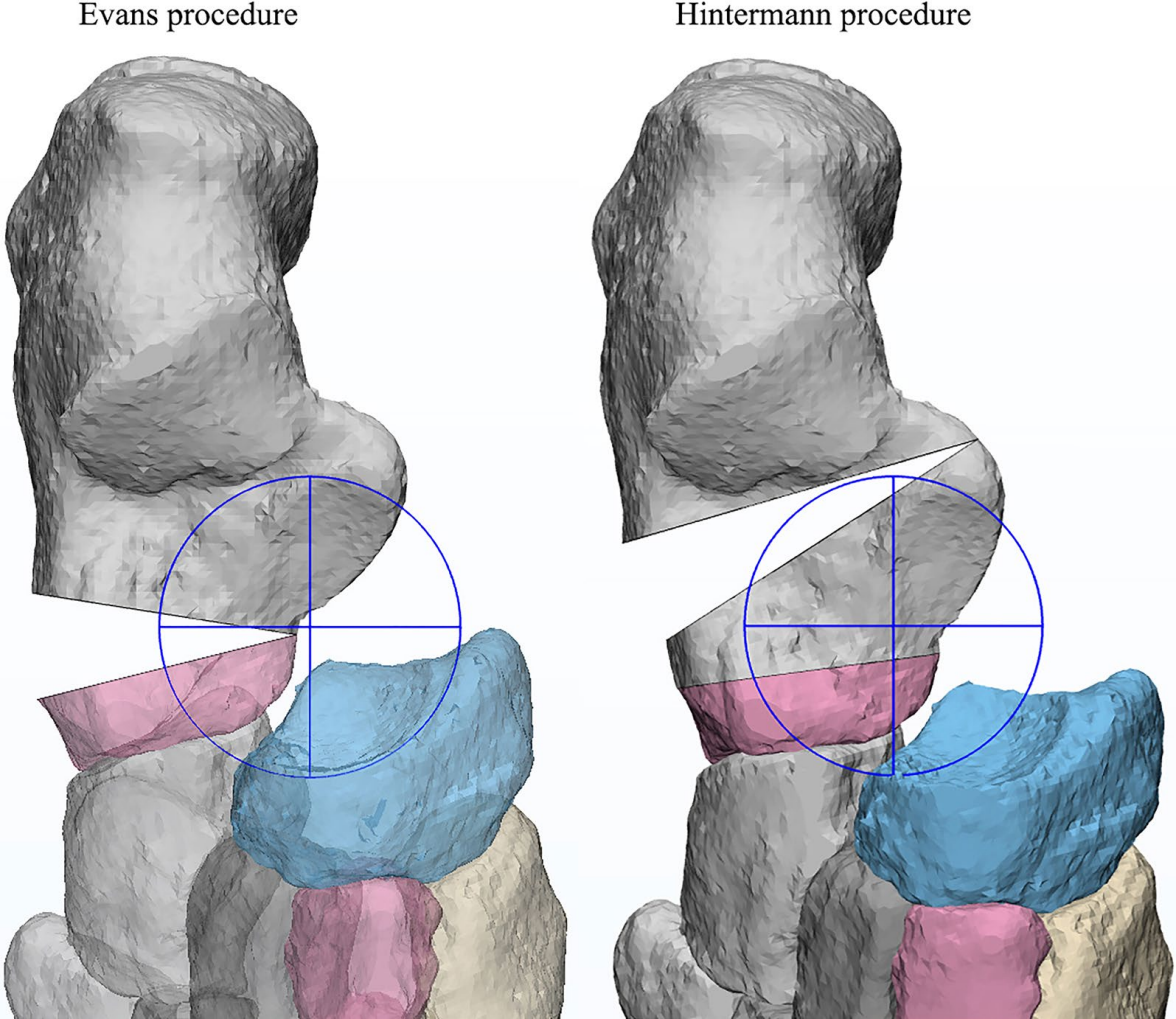
Internal rotation of the mid and forefoot based on the rotation center of both procedures

As a matter of fact, the results of our study indicate that the contact stress characteristics of the Chopart and subtalar joints in the Hintermann procedure appeared to be more abnormal than those in Evans. When using grafts of the same size, the peak pressures of the CC and TN joints in the Hintermann group were higher than those of Evans (Fig. 6a, b). In addition, the pressures of the posterior subtalar and TN joints were less evenly distributed in the Hintermann group than in the Evans group (Figs. 7, 9), and the TN joint rotation center seems to play a key role in this phenomenon. During a LCL surgery, the medial calcaneal cortex needs to be properly secured and is used as the rotation center of osteotomy. The closer this rotation center is to the TN joint rotation axis, the easier the correction of forefoot abduction will be. In this numerical study, the rotation center of the TN joint was fit by MIMICS, as shown in Fig. 10. It can be seen that the rotation center of the TN joint was very close to that of Evans osteotomy. Therefore, as the graft size increased, the navicular articular surface could still maintain a proper alignment in the Evans procedure (Fig. 11a), but a severe dislocation of TN joint would occur in the Hintermann procedure (Fig. 11b), leading to a time-consuming process to search for a new biomechanical equilibrium state in Abaqus.

The stress distribution and peak pressure of the TN joint also verified this hypothesis (Figs. 6b, 7). The average peak pressure in the Hintermann procedure was higher than that of Evans when using grafts of the same size (Fig. 6b). With the increase in graft size, the stress center of the TN joint gradually shifted inwards and downwards (Fig. 7). The decrease was slower and the contact area was relatively smaller in the Hintermann group, which may be due to the TN joint mismatch caused by the Hintermann procedure. However, an opposite trend was observed in the posterior subtalar joint. The peak stress of the posterior subtalar joint in the Hintermann group was lower than that in the Evans group (Figs. 6c, 9). A possible reason may be that the whole anterior and medial joint of subtalar joint was internally rotated during the Hintermann surgery, and a better support was provided to the head of talus, therefore resulting in a relatively lower stress force on the posterior subtalar articular surface. Considering that the Hintermann procedure was performed between the middle and posterior facets, thus imposing a lower injury risk of the subtalar joint, the Hintermann procedure can indeed provide better protection for the subtalar joint.

It has been well established that PF, SL and plantar ligament play an important role in maintaining the stability of the foot arch [27, 28]. The strain of SMCN ligament and the medial part of PF exhibited a declining trend as the graft size increased, which is consistent with previous studies [20, 27]. This is probably attributed to a reduced load on the ligaments due to the support provided by cuboid and navicular [29]. The strain of the SMCN ligament was very low when using a large graft, which means that the attenuated SL is very loose. Therefore, it may be necessary to reef and strengthen the SL during the LCL procedure in order to provide additional support to the talar head and the medial longitudinal arch.

We also observed an increased strain in the lateral bands of the PF in both groups, which may be attributed to the lengthening of the lateral column. Smith et al. [20] found that, when the strain in each fascia band remained over 6%, it could lead to rupture or degenerative changes of the ligaments. The rupture of the lateral part of ligaments could further result in lateral column pain [25]. In this study, the strain of the lateral part of PF was basically lower than 6% except for the condition when the graft length reached 14 mm in the Hintermann group. Therefore, the strain of the lateral part of PF is reliably within the safe range as long as the graft length is less than 10 mm.

Generally speaking, neither Hintermann nor Evans procedures are perfect. Hintermann procedure can provide better protection to the subtalar joint, but its disturbance to the biomechanics of Chopart joint remains a severe problem. Evans produces less disturbance to the contact characteristics of the Chopart joint. However, its incidence of fused anteromedial facets of the subtalar joint is reported to range 56–65% [30–32], and it will inevitably destroy the anterior subtalar joint of the patients. Interestingly, Evans’ original description of his procedure did not mention about the anterior or middle facet violation. He performed the osteotomy at a position about 1.5 cm from the CC joint and did not deliberately expose the space between the anterior and middle subtalar joints for the purpose of protecting the joint surface. According to several later anatomical studies [32–35], many of such surgeries in the first series must have invaded the middle facet. However, Phillips [36] reported that, in 17 of the 23 original patients, the results were satisfactory in a mean follow-up period of 13 years. Therefore, if these facet injuries have no noticeable effect on the clinical outcome, the Evans procedure should be a safer option, which of course needs further clinical studies with long-term follow-up to confirm.

Several limitations to this study are worth noting. First, our model was based on some simplifying conditions, and the material properties of ligaments were derived from literature rather than actual measurements. Despite these simplifications, the validation test showed that our simulation results were very close to the experimental measurements in previous studies. Second, the loading model was a simulation of mid-term standing posture and was not able to reflect the effect of LCL on the entire gait cycle. Third, computer simulation settings allowed for idealized placement of grafts, so the possible dislocation of proximal calcaneus tuberosity and the impact of fixation method during actual surgery were not taken into account during simulation, which may both affect the clinical outcomes. Fourth, we considered only one direction of Hintermann osteotomy. The influence of individual differences of 3D morphology and facet orientation of calcaneal on the osteotomy direction was not considered [37].

The present FE study indicates that both Evans and Hintermann procedures have good corrective ability for AAFD. The Hintermann procedure provides a stronger corrective effect than Evans, but can cause greater disturbance to the biomechanics of the Chopart joints, which may be an important mechanism of arthritis. Further clinical research with a long-term follow-up is needed to compare these two procedures more comprehensively.

### Supplementary Information

**Additional file 1.** **Table 1.** Tibia, fibula and ankle ligament properties. **Table 2.** Calcaneus, talus and midfoot bone ligament properties. **Table 3.** Planter fascia and long/short planter ligament properties. **Table 4.** The arch height for normal, AAFD populations and our model. **Table 5.** The angle measurement for normal, AAFD populations and our model.

## Abbreviations

LCL: Lateral column lengthening
AAFD: Adult acquired flatfoot deformity
FE: Finite element
TN: Talonavicular
CC: Calcaneocuboid
SL: Spring ligament
PF: Planter fascia
SMCN: Superomedial calcaneonavicular
Tal-1MA: Talus-first metatarsal angle
AP: Anteroposterior

## References

[1] Thordarson DB, Schon LC, de Cesar NC, Deland JT, Ellis SJ, Johnson JE (2020). Consensus for the indication of lateral column lengthening in the treatment of progressive collapsing foot deformity. Foot Ankle Int.

[2] Chan JY, Greenfield ST, Soukup DS, Do HT, Deland JT, Ellis SJ (2015). Contribution of lateral column lengthening to correction of forefoot abduction in stage IIb adult acquired flatfoot deformity reconstruction. Foot Ankle Int.

[3] Ettinger S, Mattinger T, Stukenborg-Colsman C, Yao D, Claassen L, Daniilidis K (2019). Outcomes of Evans versus Hintermann calcaneal lengthening osteotomy for flexible flatfoot. Foot Ankle Int.

[4] Modha RK, Kilmartin TE (2021). Lateral column lengthening for flexible adult acquired flatfoot: systematic review and meta-analysis. J Foot Ankle Surg.

[5] Ettinger S, Sibai K, Stukenborg-Colsman C, Yao D, Claassen L, Daniilidis K (2018). Comparison of anatomic structures at risk with 2 lateral lengthening calcaneal osteotomies. Foot Ankle Int.

[6] Hintermann B, Valderrabano V, Kundert HP (1999). Lateral column lenghtening by calcaneal osteotomy combined with soft tissue reconstruction for treatment of severe posterior tibial tendon dysfunction. Technique and preliminary results. Orthopade.

[7] Hintermann B, Valderrabano V, Kundert HP (1999). Lengthening of the lateral column and reconstruction of the medial soft tissue for treatment of acquired flatfoot deformity associated with insufficiency of the posterior tibial tendon. Foot Ankle Int.

[8] Harris MC, Hedrick BN, Zide JR, Thomas DM, Shivers C, Siebert MJ (2021). Effect of lateral column lengthening on subtalar motion in a cadaveric model. Foot Ankle Int.

[9] Pfeiffer FM (2016). The use of finite element analysis to enhance research and clinical practice in orthopedics. J Knee Surg.

[10] Wu J, Liu H, Xu C (2022). Biomechanical effects of graft shape for the Evans lateral column lengthening procedure: a patient-specific finite element investigation. Foot Ankle Int.

[11] Li W, Anderson DD, Goldsworthy JK, Marsh JL, Brown TD (2008). Patient-specific finite element analysis of chronic contact stress exposure after intraarticular fracture of the tibial plafond. J Orthop Res.

[12] Spratley EM, Matheis EA, Hayes CW, Adelaar RS, Wayne JS (2013). Validation of a population of patient-specific adult acquired flatfoot deformity models. J Orthop Res.

[13] Deland JT, Asla RJ, Sung I-H, Ernberg LA, Potter HG (2005). Posterior tibial tendon insufficiency: Which ligaments are involved?. Foot Ankle Int.

[14] Cifuentes-De la Portilla C, Larrainzar-Garijo R, Bayod J (2019). Analysis of the main passive soft tissues associated with adult acquired flatfoot deformity development: a computational modeling approach. J Biomech.

[15] Golano P, Vega J, de Leeuw PA, Malagelada F, Manzanares MC, Gotzens V (2010). Anatomy of the ankle ligaments: a pictorial essay. Knee Surg Sports Traumatol Arthrosc.

[16] Campbell ST, Reese KA, Ross SD, McGarry MH, Leba TB, Lee TQ (2014). Effect of graft shape in lateral column lengthening on tarsal bone position and subtalar and talonavicular contact pressure in a cadaveric flatfoot model. Foot Ankle Int.

[17] Xia J, Zhang P, Yang YF, Zhou JQ, Li QM, Yu GR (2013). Biomechanical analysis of the calcaneocuboid joint pressure after sequential lengthening of the lateral column. Foot Ankle Int.

[18] Momberger N, Morgan JM, Bachus KN, West JR (2000). Calcaneocuboid joint pressure after lateral column lengthening in a cadaveric planovalgus deformity model. Foot Ankle Int.

[19] Ellis SJ, Johnson JE, Day J, de Cesar NC, Deland JT, Hintermann B (2020). Titrating the amount of bony correction in progressive collapsing foot deformity. Foot Ankle Int.

[20] Smith BA, Adelaar RS, Wayne JS (2017). Patient specific computational models to optimize surgical correction for flatfoot deformity. J Orthop Res.

[21] Femino JE, Kern A, Schumer R, Anthony C, Kruse AJ, Goetz J (2022). The effect of progressive lateral column lengthening in a novel stage II-B flatfoot cadaveric model evaluated using software-guided radiographic measurements of foot alignment. Foot Ankle Int.

[22] Malakoutikhah H, Madenci E, Latt LD (2022). The impact of ligament tears on joint contact mechanics in progressive collapsing foot deformity: a finite element study. Clin Biomech (Bristol, Avon).

[23] Koury K, Grasu B, Stein B, Abassi P, Guyton G (2019). Biomechanical comparison of hintermann and evans osteotomy for lateral column lengthening. Foot & Ankle Orthopaedics.

[24] Deland JT, Page A, Sung IH, O'Malley MJ, Inda D, Choung S (2006). Posterior tibial tendon insufficiency results at different stages. HSS J Musculoskeletal J Hospital Spec Surg.

[25] Dinucci KR, Christensen JC, Dinucci KA (2004). Biomechanical consequences of lateral column lengthening of the calcaneus: Part I: long plantar ligament strain. J Foot Ankle Surg.

[26] Mosier-LaClair S, Pomeroy G, Manoli A (2001). Operative treatment of the difficult stage 2 adult acquired flatfoot deformity. Foot Ankle Clin.

[27] Malakoutikhah H, Madenci E, Latt LD (2022). The contribution of the ligaments in progressive collapsing foot deformity: a comprehensive computational study. J Orthop Res.

[28] Huang CK, Kitaoka HB, An KN, Chao EY (1993). Biomechanical evaluation of longitudinal arch stability. Foot Ankle.

[29] Lee MS, Vanore JV, Thomas JL, Catanzariti AR, Kogler G, Kravitz SR (2005). Diagnosis and treatment of adult flatfoot. J Foot Ankle Surg.

[30] Padmanabhan R (1986). The talar facets of the calcaneus–an anatomical note. Anat Anz.

[31] Raines RA, Brage ME (1998). Evans osteotomy in the adult foot: an anatomic study of structures at risk. Foot Ankle Int.

[32] Hyer CF, Lee T, Block AJ, VanCourt R (2002). Evaluation of the anterior and middle talocalcaneal articular facets and the Evans osteotomy. J Foot Ankle Surg.

[33] Bussewitz BW, DeVries JG, Hyer CF (2013). Evans osteotomy and risk to subtalar joint articular facets and sustentaculum tali: a cadaver study. J Foot Ankle Surg.

[34] Canavese F, Dimeglio A, Bonnel F (2018). Postoperative CT-scan 3D reconstruction of the calcaneus following lateral calcaneal lengthening osteotomy for flatfoot deformity in children: Is the surgical procedure potentially associated with subtalar joint damage?. Foot Ankle Surg Off J Eur Soc Foot Ankle Surg.

[35] Wu J, Liu H, Xu C (2022). The optimal procedure for lateral column lengthening calcaneal osteotomy according to anatomical patterns of the subtalar joint: an anatomical study in the Chinese population. BMC Musculoskeletal Disord.

[36] Phillips GE (1983). A review of elongation of os calcis for flat feet. J Bone Joint Surg Br.

[37] Efrima B, Barbero A, Ramalingam K, Indino C, Maccario C, Usuelli FG (2023). Three-dimensional distance mapping to identify safe zones for lateral column lengthening. Foot Ankle Int.

