# Supplementary material for: Biomechanical effects of Evans versus Hintermann osteotomy for treating adult acquired flatfoot deformity: a patient-specific finite element investigation
Source: J Orthop Surg Res. 2024 Feb 1;19:107. doi: 10.1186/s13018-024-04584-4 (PMC10835985; doi:10.1186/s13018-024-04584-4)
Supplement: Supplementary file 1 — Additional file 1. Table 1. Tibia, fibula and ankle ligament properties. Table 2. Calcaneus, talus and midfoot bone ligament properties. Table 3. Planter fascia and long/short planter ligament properties. Table 4. The arch height for normal, AAFD populations and our model. Table 5. The angle measurement for normal, AAFD populations and our model. [file 13018_2024_4584_MOESM1_ESM.docx]

Table 1. Tibia, fibula and ankle ligament properties.

| Ligament | *a*(N) | *b* |
| --- | --- | --- |
| Anterior talofibular | 7.18 | 12.50 |
| Anterior tibiofibular | 5.52 | 22.63 |
| Anterior tibiotalar | 2.06 | 20.11 |
| Calcaneofibular | 0.20 | 49.63 |
| Posterior talofibular | 0.14 | 44.35 |
| Posterior tibiofibular | 6.87 | 20.07 |
| Posterior tibiotalar | 1.34 | 28.65 |
| Tibiocalcaneal | 0.51 | 45.99 |
| Tibionavicular | *k*=39.1N/mm |  |

Ligament properties for eight ligaments that connect the tibia and fibula with the bones of the foot and ankle are expressed as curve fit data (a and b) for an elastic force-strain response function[[1](#_ENREF_1)] (T(ε) = a(e^bε^-1)). The stiffness *k* of the tibionavicular ligament is also provided[[2](#_ENREF_2)].

Table 2. Calcaneus, talus and midfoot bone ligament properties.

| Ligament | Area(mm^2^) | Area ratio |
| --- | --- | --- |
| Anterior talocalcaneal | 14.4 | 0.229 |
| Posterior talocalcaneal | 14.96 | 0.238 |
| Lateral talocalcaneal | 6.84 | 0.109 |
| Medial talocalcanea | 14.91 | 0.237 |
| Interosseous talocalcaneal | 72.80 | 1.158 |
| Dorsal talonavicular | 35.15 | 0.559 |
| Interosseous calcaneocuboid | 72.80 | 1.158 |
| Plantar calcaneocuboid | 98.70 | 1.570 |
| Inferior calcaneonavicular | 9.23 | 0.147 |
| Superomedial calcaneonavicular | 161.00 | 2.560 |
| Dorsal cuboideonavicular | 13.10 | 0.208 |
| Plantar cuboideonavicular | 27.80 | 0.442 |
| Interosseous cuboideonavicular | 14.01 | 0.223 |

Properties for the calcaneal, talar and midfoot bone ligaments were assumed to be the same as the anterior talofibular ligament, scaled by their relative cross-sectional areas. The anterior talofibular ligament has a cross-sectional area of 62.85 mm^2^[[3](#_ENREF_3)] and areas for all other ligaments were provided by Mkandawire[[3](#_ENREF_3)] and Shin[[4](#_ENREF_4)].

Table 3. Planter fascia and long/short planter ligament properties.

| Ligament | Stiffness *k* (N/mm) |
| --- | --- |
| Plantar fascia | 203.3 |
| Long/Short plantar ligament | 75.9 |

Linear elastic stiffness values reported in the literature for the long/short plantar ligament ligaments and plantar fascia are provided[[5](#_ENREF_5), [6](#_ENREF_6)].

Table 4. The arch height for normal, AAFD populations and our model.

|  | Tal-h | Nav-h | 1CN-h | Cub-h |
| --- | --- | --- | --- | --- |
| Normal population | 47±7.0[[7](#_ENREF_7)] | 31.3±7.3[[8](#_ENREF_8)] | 19.8±2.7[[9](#_ENREF_9)] | 12±3.7[[10](#_ENREF_10)] |
| AAFD | NA | 19±6.0[[11](#_ENREF_11)] | 11.4±4.3[[9](#_ENREF_9)] | 8.8±7.2[[10](#_ENREF_10)] |
| Our model |  | 17.1±5.4 | 9.5±3.6 | 5.7±4.2 |

Table 5. The angle measurement for normal, AAFD populations and our model.

|  | Calcaneal Pitch | Tal-1MT(ML) | Tal-Cal(ML) | Tal-1MT(AP) |
| --- | --- | --- | --- | --- |
| Normal population | 19.7±6.5[[12](#_ENREF_12)] | 3.3±4.7[[12](#_ENREF_12)] | 45.1±7.6[[12](#_ENREF_12)] | 7.1±6.6[[12](#_ENREF_12)] |
| AAFD | 16.3±3.6[[9](#_ENREF_9)] | 17.5±6.4[[10](#_ENREF_10)] | 36.2±30.5[[8](#_ENREF_8)] | 16.5±14.0[[13](#_ENREF_13)] |
| Our model | 15.5±4.7 | 13.5±5.6 | 33.3±3.4 | 13.7±4.5 |

1. Funk JR, Hall GW, Crandall JR, Pilkey WD: **Linear and quasi-linear viscoelastic characterization of ankle ligaments**. *Journal of biomechanical engineering* 2000, **122**(1):15-22.

2. Siegler S, Chen J, Schneck CD: **The three-dimensional kinematics and flexibility characteristics of the human ankle and subtalar joints--Part I: Kinematics**. *Journal of biomechanical engineering* 1988, **110**(4):364-373.

3. Mkandawire C, Ledoux WR, Sangeorzan BJ, Ching RP: **Foot and ankle ligament morphometry**. *Journal of rehabilitation research and development* 2005, **42**(6):809-820.

4. Shin J, Yue N, Untaroiu CD: **A finite element model of the foot and ankle for automotive impact applications**. *Annals of biomedical engineering* 2012, **40**(12):2519-2531.

5. Kitaoka HB, Luo ZP, Growney ES, Berglund LJ, An KN: **Material properties of the plantar aponeurosis**. *Foot & ankle international* 1994, **15**(10):557-560.

6. Cheung JT, An KN, Zhang M: **Consequences of partial and total plantar fascia release: a finite element study**. *Foot & ankle international* 2006, **27**(2):125-132.

7. Saltzman CL, Nawoczenski DA, Talbot KD: **Measurement of the medial longitudinal arch**. *Archives of physical medicine and rehabilitation* 1995, **76**(1):45-49.

8. Bruyn JM, Cerniglia MW, Chaney DM: **Combination of Evans calcaneal osteotomy and STA-Peg arthroreisis for correction of the severe pes valgo planus deformity**. *The Journal of foot and ankle surgery : official publication of the American College of Foot and Ankle Surgeons* 1999, **38**(5):339-346.

9. Coughlin MJ, Kaz A: **Correlation of Harris mats, physical exam, pictures, and radiographic measurements in adult flatfoot deformity**. *Foot & ankle international* 2009, **30**(7):604-612.

10. Younger AS, Sawatzky B, Dryden P: **Radiographic assessment of adult flatfoot**. *Foot & ankle international* 2005, **26**(10):820-825.

11. van der Krans A, Louwerens JW, Anderson P: **Adult acquired flexible flatfoot, treated by calcaneocuboid distraction arthrodesis, posterior tibial tendon augmentation, and percutaneous Achilles tendon lengthening: a prospective outcome study of 20 patients**. *Acta orthopaedica* 2006, **77**(1):156-163.

12. Thomas JL, Kunkel MW, Lopez R, Sparks D: **Radiographic values of the adult foot in a standardized population**. *The Journal of foot and ankle surgery : official publication of the American College of Foot and Ankle Surgeons* 2006, **45**(1):3-12.

13. Murley GS, Menz HB, Landorf KB: **A protocol for classifying normal- and flat-arched foot posture for research studies using clinical and radiographic measurements**. *Journal of foot and ankle research* 2009, **2**:22.
